# The impact of a participatory learning and action intervention on unmet need for contraception: a cluster-randomized controlled trial in rural Bihar, India

**DOI:** 10.1186/s12978-025-02055-5

**Published:** 2025-07-01

**Authors:** Mey-Ling Sommer, Lisa Bogler, S V Subramanian, Sebastian Vollmer

**Affiliations:** 1 Helmut-Schmidt-Universität, Hamburg, Germany; 2 Georg-August-Universität Göttingen, Göttingen, Germany; 3 Harvard Center for Population and Development Studies, Cambridge, USA; 4 Department of Social and Behavioral Sciences, Harvard T.H. Chan School of Public Health, Boston, USA

**Keywords:** Unmet need, Contraception, Participatory learning and action, India, Impact evaluation

## Abstract

**Background:**

Unmet need for contraception is a persistent issue in rural India. This work evaluates the impact of *Gram Varta* – a participatory learning and action intervention employed in women’s self-help groups in rural Bihar, India – on women’s contraceptive behavior and unmet need for contraception. Trained facilitators used an active and participatory communication approach in 20 group meetings to discuss health-related topics focusing on the improvement of communities’ knowledge and practice related to health, nutrition, water, sanitation and hygiene. One of the meetings focused on family planning measures, the benefits of various measures, and a discussion on the availability of various means of contraception through the government health system. The intervention was also meant to increase women’s empowerment.

**Methods:**

*Gram Varta* was evaluated in one district of Bihar, Madhepura, with a cluster-randomized controlled trial. We used a difference-in-differences model to estimate the intention-to-treat effect of *Gram Varta* on women’s contraceptive behaviour and unmet need for contraception using a panel dataset with 972 observations collected in 2015 and 2016.

**Results:**

We find a statistically marginally significant increase in contraceptive use by 5.8 percentage points (95%-CI [0.00;0.12]) and a statistically significant reduction in unmet need for limiting childbirth by 7.2 percentage points (95%-CI [-0.14;0.00]) among women in treatment villages but no effect on spacing childbirth.

**Conclusions:**

There is indicative evidence that participatory learning and action intervention increased contraception use among women of reproductive age in rural India, but the effect on unmet need was inconsistent.

**Trial registration:**

The randomized controlled trial was registered with and a pre-analysis plan was submitted to 3ie before intervention roll-out. In addition, it was retrospectively registered in the AEA RCT Registry with the identification number AEARCTR-0004700 on September 16, 2019.

## Background

There is large heterogeneity in the contraceptive prevalence between and within states in India. The state of Bihar performs worse than the rest of the country with respect to contraceptive uptake and status of met need in contraception among currently married women [1]. Kumar & Gupta [2] assessed estimates for family planning parameters via a user-friendly web application and identified significant discrepancies at the district-level of Bihar. Besides lack of information and needs-adapted services in line with reproductive intentions [3], Mahapatra et al. [4] also identified disadvantages in family planning services among couples with male migration. Further, Sharma & Singh [5] showed that 44.8 percent of current pregnancies among young adolescents in Bihar are still unwanted emphasizing the urgency of adequate family planning services. Although recent declines in fertility have been recorded, these are predominantly due to the widespread acceptance of sterilization in India [6]. Also, increased regret of sterilization due to deficient quality of care [7] and inadequate information provision in non-governmental hospitals regarding e.g. sterilization alternatives or possible side effects [8] require all the more an improvement of family planning services to postpone childbearing. In this context, the term “unmet need” for family planning is defined as the percentage of women without child desire but who are not using contraception to avoid conception. In this study, we rely on Bradley et al. [9] who present a revised and consistent definition of unmet need from the *Demographic and Health Surveys Program*. The revised definition includes a distinction between unmet need for limiting and spacing childbearing.

The purpose of this work is to evaluate the impact of a participatory learning and action (PLA) intervention in Bihar, India, called *Gram Varta*, on family planning and unmet need for contraception among women of reproductive age. *Gram Varta* was implemented in women’s self-help groups through a cycle of pre-structured meetings and primarily aimed to improve the communities’ knowledge and practice concerning health, sanitation, nutrition, child care and family planning. While the impact of *Gram Varta* on knowledge and practices related to health, nutrition, sanitation, and hygiene is described elsewhere [10], this study focuses on family planning and unmet need for contraception.

Daniel et al. [11] implemented behavior change communication interventions in Bihar and found that information provision of reproductive health was positively correlated with contraceptive use although direct provision of contraceptives was not part of the intervention. Still, the discussion about reproductive health and contraceptive measures requires a particular sensitive approach as communication about sex and related topics are attributed little attention or are even considered taboo [12]. However, the persisting taboo of sex without reproductive intention complicates the recognition of *modern contraception* [13] which constrains the realization of women’s individual agency. Implemented through an existing structure of self-help groups and thereby set within a familiar environment, *Gram Varta* could provide an opportunity to create awareness in sexual matters, to support behavioral intentions in reproductive health and support self-reflected decision making. In this sense, we expect the transfer and implementation of knowledge and practices through *Gram Varta* to reduce the existing gap in unwanted childbearing and contraceptive uptake in rural Bihar.

## Study design and data collection

### Intervention: Gram Varta

*Gram Varta* was implemented in more than half of all districts of Bihar and accompanied by a randomized controlled trial (RCT) in one of them, Madhepura. In Madhepura, the implementing agency was the Bihar state rural livelihoods project (Jeevika), which has mobilized rural women to set up self-help groups focusing on microfinance activities since 2006 [14]. *Gram Varta* used a PLA approach in these existing village-based women’s self-help groups. PLA interventions encourage active reflection and collaborative problem-solving within communities by leveraging participatory tools. At the core of this approach was a cycle of 20 pre-structured meetings. While meetings were intended to take place bi-weekly, actual schedules differed due to each group’s preference. Jeevika trained some of their staff members in facilitating the meetings. These facilitators invited community members to the meetings and guided the self-help groups through the PLA cycle using participatory techniques, including games, picture cards, stories, and demonstrations. Meeting content related to women’s agency, attitude towards working together, service utilization, as well as health, nutrition, water, sanitation, and hygiene (HNWASH) knowledge and practices. One of the 20 meetings focused on family planning measures, the benefits of various measures, and a discussion on the availability of various means of contraception through the government health system. The intervention was also meant to increase women’s empowerment. The meetings were not only set up for self-help group members but also non-members were invited to participate in the program and spread the word to other people in the village.

In Madhepura, *Gram Varta* was implemented in 3,129 self-help groups [10], covering a population of 1,071,348 individuals according to the 2011 population census [15]. This translated to a coverage of 342 people per self-help group.

### Experimental design

Madhepura, the district in Bihar where *Gram Varta* was evaluated with an RCT, comprises 13 blocks with 443 gram panchayats (village clusters). From those, six blocks including 68 gram panchayats were purposively selected by the implementing agency, Jeevika, for program evaluation. Jeevika chose those gram panchayats where self-help groups had been active regularly for at least two years. Randomization of the treatment assignment was done at the gram panchayat level using the statistical software Stata and was stratified by block to account for heterogeneity between blocks. Thirty-four gram panchayats with 90 villages were randomly assigned to the treatment group and the remaining 34 gram panchayats with 90 villages were assigned to the control group. *Gram Varta* was implemented strictly according to treatment assignment. In the control group, self-help group meetings continued as before, while in the treatment group, the cycle of PLA meetings was implemented in the self-help groups. The distance between gram panchayats made spill-overs across gram panchayats unlikely due to the traditional gender norms and patriarchal system prevalent in the area, which prevented women from frequently traveling to other villages [16, 17]. More details regarding intervention and experimental design are provided elsewhere [10].

### Data collection

**Fig. 1:**
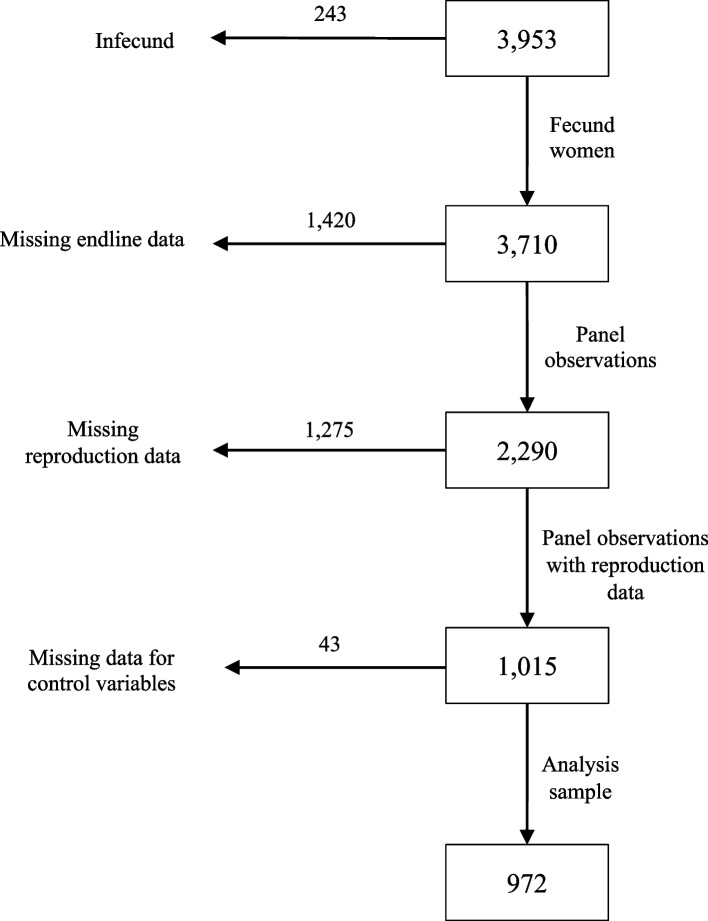
Sample selection process

*Gram Varta* meetings took place in the period from March 2015 until December 2016, six months longer than initially planned due to implementation delays during the PLA cycle. Data were collected in the selected 68 gram panchayats in three waves, at baseline (March to April 2015), midline (March 2016) and endline (November to December 2016). Based on a power calculation for other project outcomes presented elsewhere [10], a sample of 4000 households, 2000 households in control and treatment group, respectively, was planned for baseline and endline. As the midline survey was only conducted among a subset of 1000 households, it was not used for this analysis. Survey participants were randomly selected villagers and were not necessarily self-help group members or program participants. For survey sample identification, enumerators visited each sampled village and recorded the number of households in each hamlet, a subdivision of a village. Probability proportional to size of hamlet was used to determine the number of households to be interviewed in each hamlet to reach a sample of on average 22 households per village. Households were sampled through a random walk of the enumerators through the village. In each household, the household head answered questions about general information on socioeconomic and demographic characteristics and nutrition- and HNWASH-related topics. Afterwards, a woman respondent aged between 15 and 49 years with the youngest child in the household was identified. Unfortunately, the female household head, who was often older than 49 years and not the woman with the youngest child in the household, was frequently mistakenly selected as the woman respondent at baseline. Due to this misidentification, the selection criteria for the woman respondent changed from base- to mid- and endline. In order to reach the desired target group, i.e. women with children below 6 years, the wife of the oldest son of the household head with the youngest child in the household was surveyed at mid- and endline. In case the corresponding woman was not available, any married woman at reproductive age was chosen by the enumerator according to their availability. In total, 1420 out of 3953 households were affected by this change from base- to endline. The woman respondents were asked about their HNWASH practices, attitudes and beliefs, detailed information about their last-born child, whether they are members of self-help groups, knowledge about *Gram Varta* (if part of the treatment group) and topics including woman empowerment, reproductive health and social capital. Male enumerators interviewed household heads, while female enumerators interviewed woman respondents. All enumerators were trained on proper conduct during the interview as well as content of their specific survey by the research team. For the purpose of this analysis, only women who were surveyed at base- and endline, meaning those households where the woman respondent did not change due to changed selection criteria, were included in the sample to allow for a panel design. Observations with missing data in our outcome and control variables were dropped. The sample selection process is shown in Fig. 1. The final sample includes 972 woman respondent observations.

### Pre-registration and ethical clearance

The original study was initially registered with the funder before intervention roll-out and retrospectively registered in the AEA RCT Registry with the identification number AEARCTR-0004700 on September 16, 2019. Pre-registered outcomes included knowledge about contraception and child desire. Ethical clearance was provided by the Institutional Ethics Committee of the Indian Institute of Technology Gandhinagar (approval number IEC/2014–15/2/MS/006). The study was conducted in accordinace with the Declaration of Helsinki. Funding for this study was provided by the *International Initiative for Impact Evaluation* (3ie). The present study was independently compiled without involvement of 3ie.

## Methods

### Estimation strategy

A difference-in-differences estimation is applied to account for differences in pre- and post-intervention outcomes between treatment and control groups:

$${Y}_{iht} =\upbeta Di{D}_{i} + {\uplambda }_{i} + {\upgamma }_{t} + {\upvarepsilon }_{iht}$$

where *Y*_*iht*_ is the outcome for woman *i* in household *h* measured at base- and endline. *DiD*_*i*_ is an indicator variable equaling 1 for individuals being assigned to the treatment group measured after the intervention, and 0 otherwise. We measure intention-to-treat (ITT) effects as our study design does not clearly identify which survey respondent participated in the meetings. Also, we do not know who attended the particular session on family planning. The coefficient of interest β measures the ITT effects in percentage-points. λ_*i*_ and γ_*t*_ display woman- and time fixed-effects, respectively. As a robustness check, we disregard the panel structure of the data and use only endline data for an endline comparison of means. We also control for individual and socioeconomic household characteristics including the woman respondent’s age and education, whether she was married, whether she was employed in the previous 12 months, whether she was Hindu, the household size, and the block in which the household was located.^1^ We selected the control variables based on Pal et al. [18], Sharma et al. [19], and Sharma & Singh [5], who identified these factors as significant predictors of unmet need in India, particularly in Bihar, and checked for significant correlations with the outcome variables. All control variables are measured at baseline and defined as time invariant^2^; hence the control variables are omitted in the difference-in-differences estimation and only included in the endline comparison. We estimate linear probability regression models (LPM) for binary outcomes, which is a common approach for binary outcomes that are not too rare [20]. Standard errors are clustered at the gram panchayat level, the level of randomization. We assess statistical significance at the 5 percent level. Data analysis was performed using the statistical software Stata 18.

### Outcome measures

Contraceptive status and unmet need for contraception are the primary outcome variables for measuring fertility preferences. Woman respondents were first asked whether they used any method to delay pregnancy or avoid getting pregnant, followed by the question “Which method are you/your husband currently using mainly?”. The answer options included: female (male) sterilization, IUD/Copper-T, contraceptive pill, emergency contraception, injectable for women, condom or nirodh, female condom, rhythm method and withdrawal. For the analysis, only modern methods according to the definition of Hubacher & Trussell [13] are considered as active contraception. Respondents who reported practicing the rhythm method or withdrawal are treated as non-users irrespective of whether they practiced these methods successfully. The binary outcome variable *Contraception* equals 1 if any modern contraceptive method was used to avoid or delay pregnancy, and 0 otherwise. With respect to childbearing preferences, the respondents – both, pregnant and non-pregnant – were asked the following question: “[After the child you are expecting now,] Would you like to have a/another child, or would you prefer not to have any (more) children?”. Based on the questions concerning contraceptive use and child preference, the second binary outcome variable *Unmet need* is defined. The coding of the outcome variable unmet need is based on Bradley et al. [9] with the exception that it does not restrict the sample to married women only but considers all fecund women at reproductive age. It equals 1 if there is a mismatch between the use of contraceptive and stated fertility preferences, i.e. the woman respondent does not use any birth control and does not want any (more) children, and 0 otherwise. This variable equals a pooled measure for any kind of unmet need. For a more precise impact analysis of *Gram Varta*, we also distinguish between women’s unmet need for limiting and spacing childbearing depending on the woman’s child preference. Based on Nortman [21], Bradley et al. [9] classified all fecund women as having an unmet need for *limiting* childbearing if they (i) do not want to become pregnant, (ii) are currently unintentionally pregnant, or (iii) had an unwanted last birth, regardless of whether the pregnancy or birth resulted from contraceptive failure or whether they wish to have another child in the future. Women are classified as having an unmet need for *spacing* childbearing if they (i) do not want to give birth within the next two years, (ii) wanted their current pregnancy at a later time (regardless of whether it resulted from contraceptive failure) or (iii) are undecided about having a(nother) child in the future.

In more detail and in line with Bradley et al.'s [9] revised definition of unmet need, we consider women who have been married for at least five years, have not used any contraceptive method and are childless to be infecund as well.^3^ These women are excluded from the analysis, as well as those who report to have had their menopause or a hysterectomy (see Fig. 1). Further, we account for contraceptive failures by checking for current pregnancies among women who use a modern contraceptive method and do not want any children. In total, 47 women at baseline and 2 women at endline reported current pregnancies and are assigned to have an unmet need for contraception. We also check for mistimed or unintended pregnancies at endline when woman respondents reported at baseline that they wanted to limit or space childbearing.^4^ Thus, we follow Bradley et al. [9] and assign women with mistimed, unintended and also undecided current pregnancies to have an unmet need.

## Results

### Sample characteristics

Table 1 provides summary statistics for women’s characteristics by treatment and control group at baseline. The empirical strategy requires both groups to be sufficiently similar to each other with respect to individual and household characteristics, which is largely achieved by the randomized study design. In total, our sample consists of 972 unique woman observations. In this sample 69.9% of the households were Hindu with an average household size of 6.3 members. Almost half of the households (45.2%) possessed land, 13.9% used a toilet facility while only 4.3% used modern sources as fuel for cooking and 2.2% owned a car or motorcycle. The average age of the woman respondent was 30.4 years and her average age when she first gave birth was 19.4 years. Furthermore, 39.8% of the woman respondents went to school and 26.4% were employed in the previous 12 months. Almost all women could name at least one modern contraceptive method (97.8%). However, only one third (33.5%) used contraception while over two thirds (73.8%) did not want to have another child resulting in a share of 49.0% of women with an unmet need for contraception. The share of women who used contraception and had a met need was significantly larger in the control group. This difference in key outcomes at baseline necessitates the use of a panel approach for the estimation of treatment effects. There was no difference in respondents’ desire to have any more children between both groups. Combined with the significant difference in contraceptive uptake, the share of women reporting an unmet need for contraception is significantly larger in the treatment (54.7%) compared to the control group (43.6%).

**Table 1:** Sample characteristics at baseline

	Total		Control		Treatment		
	N	Mean/Share (SD)	N	Mean/Share (SD)	N	Mean/Share (SD)	Normalized difference
Age of woman (in years)	972	30.39 (7.53)	501	30.65 (7.34)	471	30.11 (7.44)	0.019
Household size (number of members)	972	6.32 (2.27)	501	6.22 (2.30)	471	6.43 (2.15)	−0.026
Share of households with land ownership	972	45.17% (61.60)	501	45.31% (63.60)	471	45.01% (58.70)	0.002
Share of households using a toilet facility	972	13.89% (47.15)	501	12.77% (44.60)	471	15.07% (49.80)	−0.017
Share of households using fuel for cooking	972	4.32% (23.59)	501	4.79% (23.22)	471	3.82% (23.88)	0.012
Share of households with a car/motorcycle	972	2.16% (15.13)	501	2.40% (12.11)	471	1.91% (18.25)	0.009
Share of households with religion ‘Hindu’	972	69.86% (55.82)	501	71.46% (57.43)	471	68.15% (52.97)	0.019
Share of married women	972	86.21% (35.76)	501	86.63% (28.28)	471	85.78% (42.35)	0.006
Share of women with employment in the previous 12 months	972	26.44% (52.92)	501	26.15% (53.55)	471	26.75% (52.97)	−0.004
Share of women with educational background	972	39.82% (49.07)	501	38.92% (46.88)	471	40.76% (50.57)	−0.010
Woman’s age when first child was born (in years)	857	19.41 (3.03)	447	19.42 (3.24)	410	19.40 (2.85)	0.002
Share of women with a child under age 6	950	38.00% (52.61)	493	36.71% (53.70)	457	39.39% (51.28)	−0.015
Share of households denoted as scheduled caste	713	89.48% (42.12)	363	91.19% (34.98)	350	87.71% (48.75)	0.035
Share of women knowing at least 1 modern contraceptive method	972	97.84% (18.20)	501	99.20% (10.21)	471	96.39% (23.24)	0.051**
Share of women using modern contraception	972	33.54% (67.92)	501	38.72% (61.01)	471	28.03% (66.50)	0.060***
Share of women who do not want another child	972	73.77% (56.20)	501	73.85% (58.53)	471	73.67% (53.23)	0.001
Share of women with unmet need	949	49.00% (59.51)	488	43.65% (59.00)	461	54.66% (47.07)	−0.059***

The table presents means of covariates at baseline for all woman respondents from the analysis sample separately for control and treatment group, as well as the difference in standard deviations between control and treatment mean. The last column reports standardized differences in means, using the average standard deviation to assess balance independently of sample size. Significance of a t-test of the normalized difference between the mean of the control and the treatment group: *** =.01, ** =.05. Standard errors are clustered at gram panchayat level. The binary variable *Share of women with educational background* equals 1 if the respondent visited at least primary school, and 0 otherwise. The binary variable on households denoted as scheduled castes equals 1 for caste categories of scheduled caste, scheduled tribe and other backward classes. The number of clusters is 68 for all variables, 34 clusters for the control and 34 for the treatment group, except household caste, which is available for 67 clusters, 33 for the control and 34 for the treatment group

Membership in self-help groups was only measured at endline. 47.4% of women in the analysis sample reported to be member in a self-help group at endline and almost all of these (93.0%) were members in a self-help group organized by Jeevika, the implementing agency of *Gram Varta* in Madhepura district. Group membership does not differ between treatment and control group (48.3% in control vs 46.5% in treatment group, *p*-value = 0.573). Among members of Jeevika self-help groups, 85.8% reported to attend regular group meetings “mostly” or “now and then”, 64.6% reported to attend “mostly”. Again, reported attendance does not differ by treatment group (83.5% vs 88.3%, *p*-value = 0.155).

Table 2 explores to what extent the analysis sample differs from the full baseline sample. In line with Bradley et al.‘s [9] definition of unmet need, the analysis sample is reduced for three reasons (see also Fig. 1): only women who are fecund are included, only women observed at baseline and endline are included, and only those with non-missing information in reproduction information and control variables are included. The sample of fecund women does not differ meaningfully from the full sample. However, among fecund women, those with panel information differ from those who dropped from the panel due to changes in the selection criteria for survey respondents. Women included in the panel are on average younger (32.8 years vs 45.4 years) and are less likely to be married (87.2% vs 93.7%) than those who dropped out. A larger share of panel women have visited school (37.1% vs 24.6%), but a smaller share was employed (26.8% vs 30.8%). The panel women with non-missing information on relevant variables again slightly differs from all panel women, notably in the share of women who use contraception (33.5% vs 50.7%). We conduct a post-hoc power analysis using G*Power [22] and run a robustness check of the analysis using the full sample of fecund women at endline, disregarding the panel structure of the data.

**Table 2:** Attrition table

	Full sample		Fecund women			Panel women		Attrited women			Women with non-missing information		
	N	Mean	N	Mean	*P*-value	N	Mean	N	Mean	*P*-value	N	Mean	*P*-value
Age of woman (in years)	3945	37.23	3702	37.59	0.0000	2286	32.78	1416	45.36	0.0000	972	30.39	0.0000
Household size (number of members)	3953	6.37	3710	6.39	0.0329	2290	6.31	1420	6.53	0.0058	972	6.32	0.8286
Share of households with land ownership	3953	47.13%	3710	46.95%	0.3917	2290	45.90%	1420	48.66%	0.1008	972	46.16%	0.5468
Share of households using a facility	3953	13.56%	3710	13.61%	0.6995	2290	12.49%	1420	15.42%	0.0113	972	13.89%	0.0857
Share of households using fuel for cooking	3953	4.53%	3710	4.42%	0.2687	2290	4.06%	1420	5.00%	0.1764	972	4.32%	0.5915
Share of households with a car/motorcycle	3953	2.73%	3710	2.56%	0.0590	2290	2.23%	1420	3.10%	0.1024	972	2.16%	0.8524
Share of households with religion ‘Hindu’	3527	70.68%	3285	70.20%	0.0129	2257	70.76%	1028	68.97%	0.2988	972	69.86%	0.4139
Share of married women	3953	89.55%	3710	89.70%	0.2640	2290	87.21%	1420	93.73%	0.0000	972	86.21%	0.2267
Share of women with employment in the previous 12 months	3953	28.11%	3710	28.30%	0.2676	2290	26.77%	1420	30.77%	0.0085	972	26.44%	0.7604
Share of women with educational background	3938	33.04%	3696	32.31%	0.0004	2283	37.10%	1413	24.56%	0.0000	972	39.81%	0.0213
Woman’s age when first child was born (in years)	3132	19.31	2942	19.31	0.9785	1833	19.40	1109	19.16	0.0170	857	19.42	0.8013
Share of women with a child under age 6	3568	19.14%	3356	19.10%	0.8010	2098	30.55%	1258	0.00%	0.0000	950	38.00%	0.0000
Share of households denoted as scheduled caste	2810	88.61%	2616	88.76%	0.3920	1718	89.06%	898	88.20%	0.5081	713	89.48%	0.6338
Share of women knowing at least 1 modern contraceptive method	3953	88.59%	3710	88.46%	0.2892	2290	88.82%	1420	87.89%	0.3870	972	97.84%	0.0000
Share of women using modern contraception	2856	50.77%	2635	49.75%	0.0002	1923	50.70%	712	47.19%	0.1095	972	33.54%	0.0000
Share of women who do not want another child	2174	78.10%	2015	77.72%	0.0919	1463	75.67%	552	83.15%	0.0003	972	73.77%	0.0145
Share of women with unmet need	2125	48.94%	1981	50.33%	0.0000	1435	48.15%	546	56.04%	0.0017	949	49.00%	0.3703

The table presents means of covariates at baseline for different sample: full sample at baseline, all fecund women, all women included in panel, all women attrited from panel, all women with non-missing information (analysis sample). *P*-values show significance of a t-test of the difference between the mean of (a) full sample and sample of fecund women, (b) panel sample and attrited sample, (c) panel sample and sample with non-missing information. The binary variable *Share of women with educational background* equals 1 if the respondent visited at least primary school, and 0 otherwise. The binary variable on households denoted as scheduled caste equals 1 for caste categories of scheduled caste, scheduled tribe and other backward classes

### Treatment effects

Table 3 presents difference-in-differences estimates for fertility outcomes as intention-to-treat effects. Living in a village cluster assigned to *Gram Varta* is associated with an increased contraceptive use by 5.8%-points (95% CI [0.00, 0.12]) which is an increase of 17.5% over the baseline value, but the coefficient is only marginally significant. The program is not statistically significantly associated with the overall probability to report an unmet need for contraception. With a decrease of 7.2%-points (95% CI [−0.14, 0.00]), there is a statistically significant association of the program with women’s unmet need for limiting childbirths. There is no statistically significant association with women’s unmet need for spacing of childbearing. Note that the sample is smaller for outcomes on unmet need for spacing as this is only estimated with women for whom this specific unmet need is defined both at baseline and endline.

**Table 3:** Intention-to-treat effects

	(1) Contraception	(2) Unmet need	(3) Unmet need for limiting	(4) Unmet need for spacing
Treatment Effect	0.058	−0.054	−0.072**	0.079
(std. error)	(0.031)	(0.041)	(0.036)	(0.096)
[95%-CI]	[0.00; 0.12]	[−0.13; 0.03]	[−0.14; 0.00]	[−0.12; 0.27]
*p-value*	*0.066*	*0.191*	*0.047*	*0.420*
Control Variables	No	No	No	No
Woman observations	972	932	603	119
Observations	1,944	1,864	1,206	238

Results of a difference-in-differences estimation with woman fixed effects. Robust standard errors and 95% confidence intervals are displayed in round and square brackets, respectively. *P*-values are displayed in italic. Significance level: ** *p* < 0.05

A post-hoc analysis of a repeated measures ANOVA (Table 4) confirmed above results, but revealed insufficient power (< 0.8) for women’s overall unmet need and unmet need for spacing with small effect sizes (Cohen’s *f* < 0.1). In the robustness check using only endline data (Table 5), we do not find any statistically significant impact on any of the outcomes. However, considering the significant baseline differences in outcome variables, we regard the difference-in-differences estimation as more reliable.

**Table 4:** ANOVA repeated measures, within-between interaction

	(1) Contraception	(2) Unmet need	(3) Unmet need for limiting	(4) Unmet need for spacing
Mean Difference	0.058**	−0.054	−0.072**	0.079
(std. error)	(0.026)	(0.038)	(0.034)	(0.098)
[95%-CI]	[0.01; 0.11]	[−0.13; 0.02]	[−0.14; −0.01]	[−0.12; 0.27]
*p-value*	*0.028*	*0.154*	*0.034*	*0.424*
F-value	4.86	2.03	4.52	0.64
Effect size (Cohen’s *f)*	0.071	0.047	0.087	0.074
Power	0.995	0.526	0.993	0.130
Woman observations	972	932	603	119
Observations	1,944	1,864	1,206	238

Results of a post-hoc analysis for repeated measures ANOVA including within-between interaction for each outcome variable. Standard errors are adjusted at the woman level to account for within-subject correlation. Mean differences are calculated based on pairwise comparisons of the interaction term with Bonferroni correction. Standard errors and 95% confidence intervals are displayed in round and square brackets, respectively. *P*-values are displayed in italic. Significance level: ** *p* < 0.05

**Table 5:** Endline comparison

	(1) Contraception		(2) Unmet need		(3) Unmet need for limiting		(4) Unmet need for spacing	
	Unadjusted	Adjusted	Unadjusted	Adjusted	Unadjusted	Adjusted	Unadjusted	Adjusted
Treatment Effect	−0.020	−0.026	0.018	0.018	0.012	0.009	0.034	0.018
(std. error.)	(0.034)	(0.029)	(0.030)	(0.026)	(0.039)	(0.034)	(0.051)	(0.048)
[95%-CI]	[−0.09; 0.05]	[−0.08; 0.03]	[−0.04; 0.08]	[−0.03; 0.07]	[−0.07; 0.09]	[−0.06; 0.08]	[−0.07; 0.14]	[−0.08; 0.11]
*p-value*	*0.561*	*0.360*	*0.540*	*0.503*	*0.760*	*0.791*	*0.510*	*0.713*
Control Variables	No	Yes	No	Yes	No	Yes	No	Yes
Woman observations	1,870	1,870	1,615	1,615	1,176	1,176	439	439

Results of a simple endline comparison of means. Standard errors and 95% confidence intervals are displayed in round and square brackets, respectively. *P*-values are displayed in italic. Unadjusted results do not include control variables. Adjusted results include the following control variables: the woman respondent’s age and education, whether she was married, whether she was employed in the previous 12 months, whether she was Hindu, household size and block

## Discussion

The PLA intervention of *Gram Varta* was established in existing women’s self-help groups with locally trained women as facilitators who guided the groups through a cycle of 20 meetings. According to the program agenda, one meeting focused on family planning. The evaluation of the intervention provided indicative evidence of an increase in contraceptive use and showed a reduction in unmet need for limiting childbirth among women.

Our findings are in line with similar intervention programs aiming to increase contraceptive use in India [23, 24], and other developing countries also by fostering couple participation [25–27]. Thus, our study contributes to the literature advocating the implementation of PLA approaches in low-resource settings to better address reproductive health needs.

We are not able to draw similar evidence on women’s overall unmet need and the unmet need for those intending to *space* childbearing. The small sub-sample size and low statistical power for the latter outcome likely influenced the results for overall unmet need. Related literature suggests that there exists a direct negative relationship between unmet need for spacing and education [28]. Given that 60% of woman respondents in our sample did not go to school, we suppose that lack of education hinders temporary birth control which is certainly more demanding in application and understanding than irreversible methods (here, sterilization). Additionally, Nayak et al. [29] found that the discontinuation rate of modern spacing methods is significantly higher in Bihar and surrounding districts. They highlight the need to shift the focus of the official family planning program, which has predominantly supported limiting (sterilization) methods and traditional approaches. Instead, they emphasize the growing need for targeted interventions.

We also support Korra’s [30] observation that spacing intentions are related to woman’s age, i.e. the share of women with reported unmet need for spacing is larger among younger woman (below 30 years) than older women while the share of women with reported unmet need for limiting does not vary strongly with age. In addition, exposure to mass media in conjunction with sufficient access to family planning services were identified to have a negative relationship with unmet need for spacing [28, 31].

This study has several limitations. First, and most importantly, Bogler et al. [10] identified constraints with respect to delays in program implementation, the short impact timeline, and the composition of meetings. Several scheduled meetings were postponed or cancelled, thus, it cannot be ensured that all treatment self-help groups actually discussed family planning topics, although it was part of the scheduled agenda. There is no data to check which meetings were held when on which topic. However, in a survey among 263 facilitators, 63% reported that contraception was discussed in every meeting, 25% reported that it was discussed often. For the estimation of the ITT effect presented in this analysis, perfect compliance is not required.

Second, contraceptive use and desired fertility are self-reported. Choi et al. [32] draw attention to potential underreporting of sterilization among populations with high sterilization rates.^5^ The responses on contraceptive use in our data confirm that there do exist mismatches between sterilizations at baseline and other reporting on contraceptive methods at endline. However, these mismatches constitute less than 2% of the endline data which may be neglected. Lastly, program implementation in existing self-help groups favors self-selection into treatment uptake for self-help group members. Still, we do not find significant differences in unmet need between members and non-members.

*Gram Varta* was designed to affect the entire community beyond self-help group members. Interested community members were invited to join the sessions even if they were not a member of a self-help group. Session topics (e.g. on family planning) were discussed in the groups and information provided by the facilitators was encouraged to be shared with family members and friends. Participation of men at *Gram Varta* meetings was low. Bhan et al. [33] find evidence for deviating perceptions and expectations regarding family planning in India and Nazarbegian et al.'s [34] highlight the important of marital communication about contraceptive use. It remains an open question if *Gram Varta* could have been more effective if it had involved husbands to strengthen spousal communication.

## Data Availability

The data necessary to replicate the study, including raw data, analysis data, and code files, are available via the repository [35] of the University of Göttingen.

## Footnotes

1 While Sharma et al. [19] highlighted the number of children and Sharma & Singh [5] and Sharma et al. [19] found caste significant, we found no significant correlation between scheduled caste and our outcome measures. We omitted variables that could be potentially influenced by the intervention (e.g. number of children under the age of 6). Additionally, data on the total number of children was unavailable, so we use household size as a proxy for family size.

2 Marital status is the only variable not fixed at baseline, as we allowed it to vary between base- and endline to capture potential changes in reproductive behavior due to a commited partnership. Including woman’s marital status as a control variable in the difference-in-differences estimation does not alter the results. In line with the revised definition by Bradley et al. [9], we also conducted a sub-group analysis including only married women to address the slight deviation in the construction of our main outcome. However, restricting the sample to married women also yields similar results, thus, these adjusted results are omitted.

3 We re-checked for the fact that women may practice successfully the rhythm method or withdrawal, however, this applies only to one woman respondent in our sample.

4 This concerns 23 and 4 women who wanted to limit and space childbearing, respectively, at baseline but were pregnant at endline. The same applies for 35 pregnant woman who were undecided whether they wanted a child in the future.

5 At baseline, 23.9% of all woman respondents reported sterilization as main contraceptive measure. Among respondents who indicated using modern contraception, this equals 82.7%.
